# Supplementary material for: The Baseline Nutritional Status Predicts Long-Term Mortality in Patients Undergoing Endovascular Therapy
Source: Nutrients. 2019 Jul 29;11(8):1745. doi: 10.3390/nu11081745 (PMC6722841; doi:10.3390/nu11081745)

Supplemental materials

SUPPLEMENTAL TABLE 1. CONUT score

Parameter	Degree of Malnutrition			
	**Normal**	**Mild**	**Moderate**	**Severe**
Albumin, g/dL	>3.5	3.0–3.49	2.5–2.9	<2.5
Score	0	2	4	6
Lymphocytes, cells/mL	>1,600	1,200–1599	800–1,199	<800
Score	0	1	2	3
Total Cholesterol, mg/dL	>180	140–179	100–139	<100
Score	0	1	2	3

SUPPLEMENTAL TABLE 2. Baseline characteristics: CLI vs. Non-CLI

Variables	Non-HD						HD						p Value Non-HD vs. HD
	Overall N = 340	Low risk CONUT 0 N = 73	Mild risk CONUT 1-2 N = 160	Moderate risk CONUT3-4 N = 71	High risk CONUT 5=< N = 36	p Value	Overall N = 288	Low risk CONUT 0 N = 8	Mild risk CONUT 1-2 N = 90	Moderate risk CONUT3-4 N = 98	High risk CONUT 5=< N = 92	p Value	
Age, years	71 ± 9	71 ± 9	72 ± 10	69 ± 9	72 ± 11	0.02	67 ± 10	61 ± 12	65 ± 11	68 ± 10	69 ± 9	0.03	< 0.001
>75 y/o	133 (39%)	22 (30%)	70 (44%)	19 (27%)	18 (50%)	0.03	72 (25%)	1 (13%)	17 (19%)	26 (27%)	28 (30%)	0.26	< 0.001
Male	226 (66%)	37 (51%)	105 (66%)	56 (79%)	28 (78%)	0.002	208 (72%)	5 (63%)	63 (70%)	68 (69%)	72 (78%)	0.45	0.14
BMI, kg/m^2	23.1 ± 3.1	23.3 ± 3.2	23.1 ± 3.0	22.8 ± 3.1	23.0 ± 3.2	0.66	23.5 ± 4.0	24.1 ± 3.9	25.0 ± 4.1	22.6 ± 3.5	22.9 ± 4.1	< 0.001	0.57
ABI	0.67 ± 0.2	0.67 ± 0.2	0.67 ± 0.2	0.68 ± 0.2	0.62 ± 0.2	0.50	0.68 ± 0.2	0.58 ± 0.3	0.70 ± 0.2	0.65 ± 0.2	0.68 ± 0.3	0.62	0.63
Hypertension	290 (85%)	56 (77%)	144 (90%)	58 (82%)	32 (89%)	0.04	230 (80%)	6 (75%)	75 (83%)	77 (79%)	72 (78%)	0.79	0.09
Diabetes	231 (68%)	48 (66%)	103 (64%)	52 (73%)	28 (78%)	0.42	214 (74%)	6 (75%)	66 (73%)	73 (74%)	69 (75%)	0.99	0.09

Dyslipidemia	268 (79%)	58 (79%)	131 (82%)	52 (73%)	27 (75%)	0.50	200 (69%)	6 (75%)	68 (76%)	63 (64%)	63 (68%)	0.40	0.008
Smoking history	129 (38%)	32 (44%)	63 (39%)	23 (32%)	11 (31%)	0.36	108 (38%)	1 (13%)	39 (43%)	32 (33%)	36 (39%)	0.21	0.93
Prior PCI	141 (41%)	25 (34%)	66 (41%)	30 (42%)	20 (56%)	0.26	164 (57%)	4 (50%)	52 (58%)	52 (53%)	56 (61%)	0.72	< 0.001
Prior CABG	44 (13%)	4 (5%)	19 (12%)	7 (10%)	14 (39%)	0.43	65 (23%)	1 (13%)	28 (31%)	21 (21%)	15 (16%)	0.10	< 0.001
CKD †	186 (55%)	35 (48%)	88 (55%)	40 (56%)	23 (64%)	0.40	288 (100%)	8 (100%)	90 (100%)	98 (100%)	92 (100%)	-	< 0.001
LVEF<40%	28 (8%)	2 (3%)	8 (5%)	10 (14%)	8 (22%)	< 0.001	45 (16%)	1 (13%)	10 (11%)	13 (13%)	21 (23%)	0.14	0.006
CLI	103 (30%)	15 (21%)	40 (25%)	28 (39%)	24 (67%)	< 0.001	160 (56%)	4 (50%)	37 (41%)	71 (72%)	76 (83%)	< 0.001	< 0.001
Rutherford classification (1-3/4/5/6)	232/25/73/10	62/1/8/2	116/13/29/2	42/6/23/0	12/5/13/6	< 0.001	127/15/139/7	4/1/3/0	53/2/34/1	54/10/32/2	16/2/70/4	< 0.001	< 0.001
Lab data													
WBC, /µl	6,795 ± 1,899	7,102 ± 1,929	6,701 ± 1,656	6,365 ± 2,085	7,438 ± 2,180	0.02	7,060 ± 3,152	8,080 ± 1,233	7,207 ± 2,384	6,238 ± 1,987	7,704 ± 4,494	0.003	0.78
Neutrophil, /µl	4,585 ± 1,703	4,410 ± 1,759	4,392 ± 1,417	4,674 ± 1,858	5,619 ± 2,084	0.01	5,111 ± 2,950	5,237 ± 1,414	4,911 ± 1,914	4,420 ± 1,796	6,032 ± 4,341	0.01	0.13
Lymphocyte, /µl	1,567 ± 577	2,053 ± 378	1,661 ± 487	1,103 ± 373	1,083 ± 599	< 0.001	1,165 ± 475	1,877 ± 259	1,479 ± 501	1,062 ± 354	905 ± 323	< 0.001	< 0.001

Hemoglobin, g/dl	12.8 ± 1.9	13.6 ± 1.4	13.0 ± 1.6	12.1 ± 2.2	11.4 ± 2.4	< 0.001	11.3 ± 1.4	12.3 ± 1.1	11.8 ±1.4	11.3 ± 1.2	10.9 ± 1.4	< 0.001	< 0.001
Albumin, mg/dl	4.0 ± 0.5	4.2 ± 0.4	4.2 ± 0.4	3.9 ± 0.4	3.0 ± 0.5	< 0.001	3.6 ± 0.5	4.0 ± 0.1	3.9 ± 0.3	3.7 ± 0.3	3.2 ± 0.5	< 0.001	< 0.001
BUN, mg/dl	20 ± 9.5	18 ± 5.5	19 ± 8.3	21 ± 10	23 ± 15	0.62	43 ± 17	52 ± 17	43 ±19	41 ± 13	44 ± 17	0.25	< 0.001
Creatinine, mg/dl	1.1 ± 0.7	0.9 ± 0.3	1.1 ± 0.9	1.1 ± 0.5	1.2 ± 0.5	0.002	7.6 ± 2.1	8.3 ± 2.0	7.9 ± 2.0	7.6 ± 2.1	7.3 ± 2.3	0.06	< 0.001
eGFR, ml/min/1.73m^2	50 ± 20	63 ± 20	57 ± 21	57 ± 21	53 ± 21	0.05	6.4 ± 2.2	5.6 ± 1.9	6.1 ± 2.3	6.3 ± 2.1	6.8 ± 2.2	0.02	< 0.001
CRP, mg/dl	1.5 ± 8.9	2.1 ± 13	0.5 ± 1.0	2.6 ± 14	3.0 ± 5.1	< 0.001	3.5 ± 11	12.8 ± 34	2.3 ± 11	1.0 ± 1.7	6.4 ± 12	< 0.001	< 0.001
BNP, pg/dl	197 ± 397	107 ± 159	102 ± 137	268 ± 444	657 ± 841	< 0.001	596 ± 776	149 ± 105	466 ± 626	545 ± 786	824 ± 879	0.001	< 0.001
Total cholesterol, mg/dl	177 ± 39	211 ± 25	177 ± 36	156 ± 37	150 ± 31	< 0.001	162 ± 40	203 ± 16	179 ± 31	158 ± 32	145 ± 47	< 0.001	< 0.001
LDL-cholesterol, mg/dl	100 ± 32	126 ± 33	98 ± 27	86 ± 26	82 ± 28	< 0.001	88 ± 30	109 ± 28	104 ± 25	87 ± 30	74 ± 25	< 0.001	< 0.001
HDL-cholesterol, mg/dl	55 ± 18	59 ± 17	56 ± 18	50 ± 14	52 ± 22	< 0.001	49 ± 14	53 ± 12	51 ± 15	50 ± 14	45 ± 14	0.01	< 0.001
Triglyceride, mg/dl	138 ± 76	167 ± 87	141 ± 77	121 ± 63	104 ± 41	< 0.001	135 ± 112	237 ± 90	139 ± 59	124 ± 71	133 ± 170	< 0.001	0.23
HbA1c, %	6.9 ± 1.2	7.0 ± 1.2	6.8 ± 1.3	6.9 ± 1.1	7.0 ± 1.6	0.50	6.5 ± 1.2	6.8 ± 1.3	6.7 ± 1.1	6.6 ± 1.5	6.4 ± 0.9	0.48	< 0.001

Target vessel													
Aorto-iliac	71 (21%)	15 (21%)	35 (22%)	17 (24%)	4 (11%)	0.42	40 (14%)	2 (25%)	15 (17%)	12 (12%)	11 (12%)	0.59	0.027
Femoro-popliteal	209 (61%)	46 (63%)	106 (66%)	36 (51%)	21 (58%)	0.17	140 (49%)	3 (38%)	49 (54%)	60 (61%)	28 (30%)	< 0.001	0.001
Below the knee	115 (34%)	18 (25%)	49 (31%)	26 (37%)	22 (61%)	0.002	160 (56%)	3 (38%)	38 (42%)	44 (45%)	75 (82%)	< 0.001	< 0.001
Medications													
Aspirin	192 (56%)	38 (52%)	97 (61%)	35 (49%)	22 (61%)	0.31	207 (72%)	7 (88%)	70 (78%)	67 (68%)	63 (68%)	0.31	< 0.001
Thienopyridine	191 (56%)	37 (51%)	92 (58%)	42 (59%)	20 (56%)	0.71	165 (57%)	3 (38%)	50 (56%)	61 (62%)	51 (55%)	0.48	0.81
Cilostazole	92 (27%)	13 (18%)	53 (33%)	18 (25%)	8 (22%)	0.11	61 (21%)	1 (13%)	20 (22%)	24 (24%)	16 (17%)	0.61	0.09
OAC	63 (19%)	14 (19%)	24 (15%)	16 (23%)	9 (25%)	0.42	39 (14%)	2 (25%)	7 (8%)	15 (15%)	15 (16%)	0.23	0.10
ACE inhibitors or ARBs	227 (67%)	45 (62%)	115 (72%)	45 (63%)	22 (61%)	0.23	129 (45%)	3 (38%)	37 (41%)	45 (46%)	44 (48%)	0.79	< 0.001
β Blockers	110 (32%)	59 (81%)	50 (31%)	27 (38%)	18 (50%)	0.02	163 (57%)	4 (50%)	54 (60%)	49 (50%)	56 (61%)	0.40	< 0.001
Statins	205 (60%)	43 (59%)	96 (60%)	42 (59%)	24 (67%)	0.82	125 (43%)	5 (63%)	34 (38%)	45 (46%)	41 (45%)	0.45	< 0.001

ACE = angiotensin converting enzyme; ARB = angiotensin II receptor blocker; BNP = brain natriuretic peptide; BUN = blood urea nitrogen; CLI = critical limb ischemia; CKD = chronic kidney disease; CRP = C-reactive protein; DM = diabetes mellitus; eGFR = estimated glomerular filtration rate; HbA1c = hemoglobin A1c; LDL = low-density lipoprotein; LVEF = left ventricular ejection fraction; OAC = oral anticoagulant; WBC = white blood cell. * Body mass index >25 kg/m2; † eGFR <60 mL/min/1.73m^2

SUPPLEMENTAL TABLE 3. Baseline characteristics: HD vs. Non-HD

Variables	Non-HD						HD						p Value
	Overall<br>N = 340	Low risk CONUT 0<br>N = 73	Mild risk CONUT 1-2<br>N = 160	Moderate risk CONUT3-4<br>N = 71	High risk CONUT 5=<<br>N = 36	p Value	Overall<br>N = 288	Low risk CONUT 0<br>N = 8	Mild risk CONUT 1-2<br>N = 90	Moderate risk CONUT3-4<br>N = 98	High risk CONUT 5=<<br>N = 92	p Value	Non-HD vs. HD
Age, years	71 ± 9	71 ± 9	72 ± 10	69 ± 9	72 ± 11	0.02	67 ± 10	61 ± 12	65 ± 11	68 ± 10	69 ± 9	0.03	< 0.001
>75 y/o	133 (39%)	22 (30%)	70 (44%)	19 (27%)	18 (50%)	0.03	72 (25%)	1 (13%)	17 (19%)	26 (27%)	28 (30%)	0.26	< 0.001
Male	226 (66%)	37 (51%)	105 (66%)	56 (79%)	28 (78%)	0.002	208 (72%)	5 (63%)	63 (70%)	68 (69%)	72 (78%)	0.45	0.14
BMI, kg/m^2	23.1 ± 3.1	23.3 ± 3.2	23.1 ± 3.0	22.8 ± 3.1	23.0 ± 3.2	0.66	23.5 ± 4.0	24.1 ± 3.9	25.0 ± 4.1	22.6 ± 3.5	22.9 ± 4.1	< 0.001	0.57
ABI	0.67 ± 0.2	0.67 ± 0.2	0.67 ± 0.2	0.68 ± 0.2	0.62 ± 0.2	0.50	0.68 ± 0.2	0.58 ± 0.3	0.70 ± 0.2	0.65 ± 0.2	0.68 ± 0.3	0.62	0.63
Hypertension	290 (85%)	56 (77%)	144 (90%)	58 (82%)	32 (89%)	0.04	230 (80%)	6 (75%)	75 (83%)	77 (79%)	72 (78%)	0.79	0.09
Diabetes	231 (68%)	48 (66%)	103 (64%)	52 (73%)	28 (78%)	0.42	214 (74%)	6 (75%)	66 (73%)	73 (74%)	69 (75%)	0.99	0.09

Dyslipidemia	268 (79%)	58 (79%)	131 (82%)	52 (73%)	27 (75%)	0.50	200 (69%)	6 (75%)	68 (76%)	63 (64%)	63 (68%)	0.40	0.008
Smoking history	129 (38%)	32 (44%)	63 (39%)	23 (32%)	11 (31%)	0.36	108 (38%)	1 (13%)	39 (43%)	32 (33%)	36 (39%)	0.21	0.93
Prior PCI	141 (41%)	25 (34%)	66 (41%)	30 (42%)	20 (56%)	0.26	164 (57%)	4 (50%)	52 (58%)	52 (53%)	56 (61%)	0.72	< 0.001
Prior CABG	44 (13%)	4 (5%)	19 (12%)	7 (10%)	14 (39%)	0.43	65 (23%)	1 (13%)	28 (31%)	21 (21%)	15 (16%)	0.10	< 0.001
CKD †	186 (55%)	35 (48%)	88 (55%)	40 (56%)	23 (64%)	0.40	288 (100%)	8 (100%)	90 (100%)	98 (100%)	92 (100%)	-	< 0.001
LVEF<40%	28 (8%)	2 (3%)	8 (5%)	10 (14%)	8 (22%)	< 0.001	45 (16%)	1 (13%)	10 (11%)	13 (13%)	21 (23%)	0.14	0.006
CLI	103 (30%)	15 (21%)	40 (25%)	28 (39%)	24 (67%)	< 0.001	160 (56%)	4 (50%)	37 (41%)	71 (72%)	76 (83%)	< 0.001	< 0.001
Rutherford classification (1-3/4/5/6)	232/25/73/10	62/1/8/2	116/13/29/2	42/6/23/0	12/5/13/6	< 0.001	127/15/139/7	4/1/3/0	53/2/34/1	54/10/32/2	16/2/70/4	< 0.001	< 0.001
Lab data													
WBC, /µl	6,795 ± 1,899	7,102 ± 1,929	6,701 ± 1,656	6,365 ± 2,085	7,438 ± 2,180	0.02	7,060 ± 3,152	8,080 ± 1,233	7,207 ± 2,384	6,238 ± 1,987	7,704 ± 4,494	0.003	0.78
Neutrophil, /µl	4,585 ± 1,703	4,410 ± 1,759	4,392 ± 1,417	4,674 ± 1,858	5,619 ± 2,084	0.01	5,111 ± 2,950	5,237 ± 1,414	4,911 ± 1,914	4,420 ± 1,796	6,032 ± 4,341	0.01	0.13
Lymphocyte, /µl	1,567 ± 577	2,053 ± 378	1,661 ± 487	1,103 ± 373	1,083 ± 599	< 0.001	1,165 ± 475	1,877 ± 259	1,479 ± 501	1,062 ± 354	905 ± 323	< 0.001	< 0.001

Hemoglobin, g/dl	12.8 ± 1.9	13.6 ± 1.4	13.0 ± 1.6	12.1 ± 2.2	11.4 ± 2.4	< 0.001	11.3 ± 1.4	12.3 ± 1.1	11.8 ±1.4	11.3 ± 1.2	10.9 ± 1.4	< 0.001	< 0.001
Albumin, mg/dl	4.0 ± 0.5	4.2 ± 0.4	4.2 ± 0.4	3.9 ± 0.4	3.0 ± 0.5	< 0.001	3.6 ± 0.5	4.0 ± 0.1	3.9 ± 0.3	3.7 ± 0.3	3.2 ± 0.5	< 0.001	< 0.001
BUN, mg/dl	20 ± 9.5	18 ± 5.5	19 ± 8.3	21 ± 10	23 ± 15	0.62	43 ± 17	52 ± 17	43 ±19	41 ± 13	44 ± 17	0.25	< 0.001
Creatinine, mg/dl	1.1 ± 0.7	0.9 ± 0.3	1.1 ± 0.9	1.1 ± 0.5	1.2 ± 0.5	0.002	7.6 ± 2.1	8.3 ± 2.0	7.9 ± 2.0	7.6 ± 2.1	7.3 ± 2.3	0.06	< 0.001
eGFR, ml/min/1.73m^2	50 ± 20	63 ± 20	57 ± 21	57 ± 21	53 ± 21	0.05	6.4 ± 2.2	5.6 ± 1.9	6.1 ± 2.3	6.3 ± 2.1	6.8 ± 2.2	0.02	< 0.001
CRP, mg/dl	1.5 ± 8.9	2.1 ± 13	0.5 ± 1.0	2.6 ± 14	3.0 ± 5.1	< 0.001	3.5 ± 11	12.8 ± 34	2.3 ± 11	1.0 ± 1.7	6.4 ± 12	< 0.001	< 0.001
BNP, pg/dl	197 ± 397	107 ± 159	102 ± 137	268 ± 444	657 ± 841	< 0.001	596 ± 776	149 ± 105	466 ± 626	545 ± 786	824 ± 879	0.001	< 0.001
Total cholesterol, mg/dl	177 ± 39	211 ± 25	177 ± 36	156 ± 37	150 ± 31	< 0.001	162 ± 40	203 ± 16	179 ± 31	158 ± 32	145 ± 47	< 0.001	< 0.001
LDL-cholesterol, mg/dl	100 ± 32	126 ± 33	98 ± 27	86 ± 26	82 ± 28	< 0.001	88 ± 30	109 ± 28	104 ± 25	87 ± 30	74 ± 25	< 0.001	< 0.001
HDL-cholesterol, mg/dl	55 ± 18	59 ± 17	56 ± 18	50 ± 14	52 ± 22	< 0.001	49 ± 14	53 ± 12	51 ± 15	50 ± 14	45 ± 14	0.01	< 0.001
Triglyceride, mg/dl	138 ± 76	167 ± 87	141 ± 77	121 ± 63	104 ± 41	< 0.001	135 ± 112	237 ± 90	139 ± 59	124 ± 71	133 ± 170	< 0.001	0.23
HbA1c, %	6.9 ± 1.2	7.0 ± 1.2	6.8 ± 1.3	6.9 ± 1.1	7.0 ± 1.6	0.50	6.5 ± 1.2	6.8 ± 1.3	6.7 ± 1.1	6.6 ± 1.5	6.4 ± 0.9	0.48	< 0.001

Target vessel													
Aorto-iliac	71 (21%)	15 (21%)	35 (22%)	17 (24%)	4 (11%)	0.42	40 (14%)	2 (25%)	15 (17%)	12 (12%)	11 (12%)	0.59	0.027
Femoro-popliteal	209 (61%)	46 (63%)	106 (66%)	36 (51%)	21 (58%)	0.17	140 (49%)	3 (38%)	49 (54%)	60 (61%)	28 (30%)	< 0.001	0.001
Below the knee	115 (34%)	18 (25%)	49 (31%)	26 (37%)	22 (61%)	0.002	160 (56%)	3 (38%)	38 (42%)	44 (45%)	75 (82%)	< 0.001	< 0.001
Medications													
Aspirin	192 (56%)	38 (52%)	97 (61%)	35 (49%)	22 (61%)	0.31	207 (72%)	7 (88%)	70 (78%)	67 (68%)	63 (68%)	0.31	< 0.001
Thienopyridine	191 (56%)	37 (51%)	92 (58%)	42 (59%)	20 (56%)	0.71	165 (57%)	3 (38%)	50 (56%)	61 (62%)	51 (55%)	0.48	0.81
Cilostazole	92 (27%)	13 (18%)	53 (33%)	18 (25%)	8 (22%)	0.11	61 (21%)	1 (13%)	20 (22%)	24 (24%)	16 (17%)	0.61	0.09
OAC	63 (19%)	14 (19%)	24 (15%)	16 (23%)	9 (25%)	0.42	39 (14%)	2 (25%)	7 (8%)	15 (15%)	15 (16%)	0.23	0.10
ACE inhibitors or ARBs	227 (67%)	45 (62%)	115 (72%)	45 (63%)	22 (61%)	0.23	129 (45%)	3 (38%)	37 (41%)	45 (46%)	44 (48%)	0.79	< 0.001
β Blockers	110 (32%)	59 (81%)	50 (31%)	27 (38%)	18 (50%)	0.02	163 (57%)	4 (50%)	54 (60%)	49 (50%)	56 (61%)	0.40	< 0.001
Statins	205 (60%)	43 (59%)	96 (60%)	42 (59%)	24 (67%)	0.82	125 (43%)	5 (63%)	34 (38%)	45 (46%)	41 (45%)	0.45	< 0.001

ACE = angiotensin converting enzyme; ARB = angiotensin II receptor blocker; BNP = brain natriuretic peptide; BUN = blood urea nitrogen; CLI = critical limb ischemia; CKD = chronic kidney disease; CRP = C-reactive protein; DM = diabetes mellitus; eGFR = estimated glomerular filtration rate; HbA1c = hemoglobin A1c; LDL = low-density lipoprotein; LVEF = left ventricular ejection fraction; OAC = oral anticoagulant; WBC = white blood cell. * Body mass index >25 kg/m2; † eGFR <60 mL/min/1.73m^2.

SUPPLEMENTAL TABLE 4. Cause of death

	ALL<br>n = 628	non-CLI<br>n = 365	CLI<br>n = 263	p Value
Total	95 (15.1%)	35 (9.6%)	60 (22.8%)	< 0.001
Sudden death	15 (2.3%)	2 (0.5%)	13 (4.9%)	< 0.001
Sepsis	27 (4.3%)	9 (2.4%)	18 (6.8%)	0.007
Heart failure	5 (0.8%)	2 (0.5%)	3 (1.1%)	0.41
AMI	3 (0.5%)	2 (0.5%)	1 (0.4%)	0.77
Pneumoniae	8 (1.3%)	1 (0.3%)	7 (2.7%)	0.008
Renal failure	5 (0.8%)	0	5 (1.9%)	0.008
Hemorrhage	1 (0.2%)	1 (0.3%)	0	0.40
Others	31 (4.9%)	18 (4.9%)	13 (4.9%)	0.99

Supplemental figure legend

SUPPLEMENTAL FIGURE 1. Major amputation after Endovascular Therapy in Patients with Critical Limb Ischemia.

Kaplan-Meier curves of the major amputation rates for the 3 subgroups categorized according to the CONUT score on admission in CLI patients. CONUT = Controlling Nutritional Status score; EVT = endovascular therapy.

SUPPLEMENTAL FIGURE 2. All-cause Mortality After Endovascular Therapy in Patients Who Did and Did Not Undergo Hemodialysis

Kaplan-Meier curves of the **all-cause mortality** rates within the 4 subgroups categorized according to the CONUT score on admission in **(A)** non-hemodialysis patients and **(B)** hemodialysis patients. CONUT = Controlling Nutritional Status score; EVT = endovascular therapy; HD = hemodialysis.

SUPPLEMENTAL FIGURE 3. Combined Outcome of Death from Any Cause and Major Amputation After Endovascular Therapy in Patients in Relation to Each CONUT Score Component.

Supplemental figure

Supplemental Figure 1

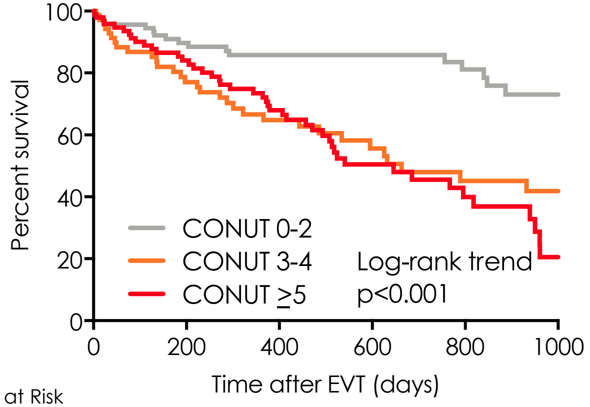

No. of Pts at Risk

CONUT 0-2	91	72	61	46	34	19
CONUT 3-4	71	48	35	23	17	14
CONUT ≥5	99	67	47	24	15	6

Supplemental Figure 2

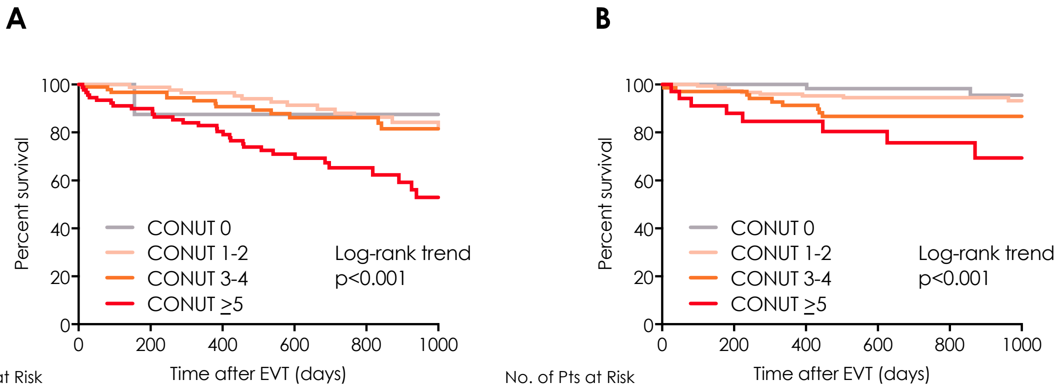

A — No. of Pts at Risk

	0	200	400	600	800	1000
CONUT 0	8	8	8	7	5	5
CONUT 1-2	90	87	81	65	50	34
CONUT 3-4	98	87	72	52	41	33
CONUT ≥5	92	77	65	44	24	14

B — No. of Pts at Risk

	0	200	400	600	800	1000
CONUT 0	73	69	60	46	38	26
CONUT 1-2	160	147	135	117	93	72
CONUT 3-4	71	68	62	51	40	32
CONUT ≥5	36	28	23	18	13	8

Supplemental Figure 3

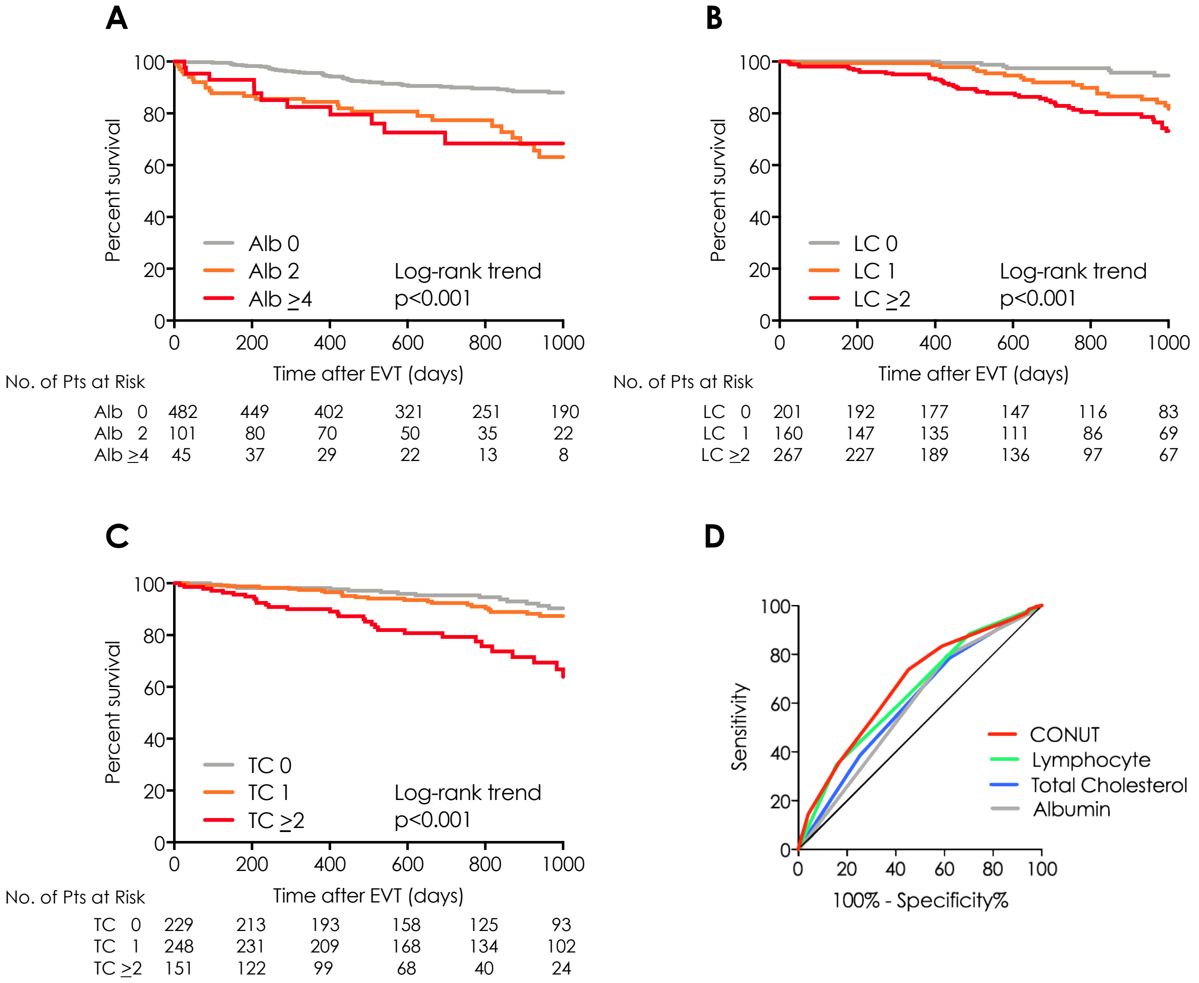

Supplement: Supplementary file 1 [file nutrients-11-01745-s001.pdf]
